# Crystal structure of K[Hg(SCN)_3_] – a redetermination

**DOI:** 10.1107/S1600536814013403

**Published:** 2014-08-01

**Authors:** Matthias Weil, Thomas Häusler

**Affiliations:** a Institute for Chemical Technologies and Analytics, Division of Structural Chemistry, Vienna University of Technology, Getreidemarkt 9/164-SC, A-1060 Vienna, Austria

**Keywords:** crystal structure, redetermination, phase transition, mercury

## Abstract

The crystal structure of the room-temperature modification of K[Hg(SCN)_3_], potassium tri­thio­cyanato­mercurate(II), was redetermined based on modern CCD data. In comparison with the previous report [Zhdanov & Sanadze (1952 ▶). *Zh. Fiz. Khim.* **26**, 469–478], reliability factors, standard deviations of lattice parameters and atomic coordinates, as well as anisotropic displacement parameters, were revealed for all atoms. The higher precision and accuracy of the model is, for example, reflected by the Hg—S bond lengths of 2.3954 (11), 2.4481 (8) and 2.7653 (6) Å in comparison with values of 2.24, 2.43 and 2.77 Å. All atoms in the crystal structure are located on mirror planes. The Hg^2+^ cation is surrounded by four S atoms in a seesaw shape [S—Hg—S angles range from 94.65 (2) to 154.06 (3)°]. The HgS_4_ polyhedra share a common S atom, building up chains extending parallel to [010]. All S atoms of the resulting ^1^_∞_[HgS_2/1_S_2/2_] chains are also part of SCN^−^ anions that link these chains with the K^+^ cations into a three-dimensional network. The K—N bond lengths of the distorted KN_7_ polyhedra lie between 2.926 (2) and 3.051 (3) Å.

## Related literature   

K[Hg(SCN)_3_] has been determined originally in the space group *P*2_1_/*m* with *Z* = 8, based on room-temperature data (Zhdanov & Sanadze, 1952 ▶). A subsequent redetermination revealed a doubled unit cell in *P*2_1_/*n*, *Z* = 4, based on intensity data measured at 150 K (Bowmaker *et al.*, 1998 ▶). However, there is no report on an apparent phase transition of K[Hg(SCN)_3_] between these two temperatures. For symmetry relationships between crystal structures, see: Müller (2013 ▶).

## Experimental   

### Crystal data  

K[Hg(NCS)_3_]
*M* *_r_* = 413.93
Monoclinic, 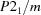

*a* = 11.2727 (11) Å
*b* = 4.0775 (4) Å
*c* = 10.9764 (10) Å
β = 114.951 (4)°
*V* = 457.44 (8) Å^3^
*Z* = 2
Mo *K*α radiation
μ = 17.90 mm^−1^
*T* = 293 K
0.30 × 0.06 × 0.04 mm

### Data collection  

Bruker APEXII CCD diffractometer
Absorption correction: multi-scan (*SADABS*; Bruker, 2008 ▶) *T*_min_ = 0.226, *T*_max_ = 0.504
11940 measured reflections
3765 independent reflections
2358 reflections with *I* > 2σ(*I*)
*R*_int_ = 0.032

### Refinement  

*R*[*F* ^2^ > 2σ(*F* ^2^)] = 0.028
*wR*(*F* ^2^) = 0.062
*S* = 1.03
3765 reflections
68 parameters
Δρ_max_ = 1.27 e Å^−3^
Δρ_min_ = −1.33 e Å^−3^

### 

Data collection: *APEX2* (Bruker, 2008 ▶); cell refinement: *SAINT* (Bruker, 2008 ▶); data reduction: *SAINT*; program(s) used to solve structure: *SHELXS97* (Sheldrick, 2008 ▶); program(s) used to refine structure: *SHELXL97* (Sheldrick, 2008 ▶); molecular graphics: *ATOMS for Windows* (Dowty, 2006 ▶); software used to prepare material for publication: *publCIF* (Westrip, 2010 ▶).

## Supplementary Material

Crystal structure: contains datablock(s) I, global. DOI: 10.1107/S1600536814013403/hb0013sup1.cif

Structure factors: contains datablock(s) I. DOI: 10.1107/S1600536814013403/hb0013Isup2.hkl

**Fig. 1.**
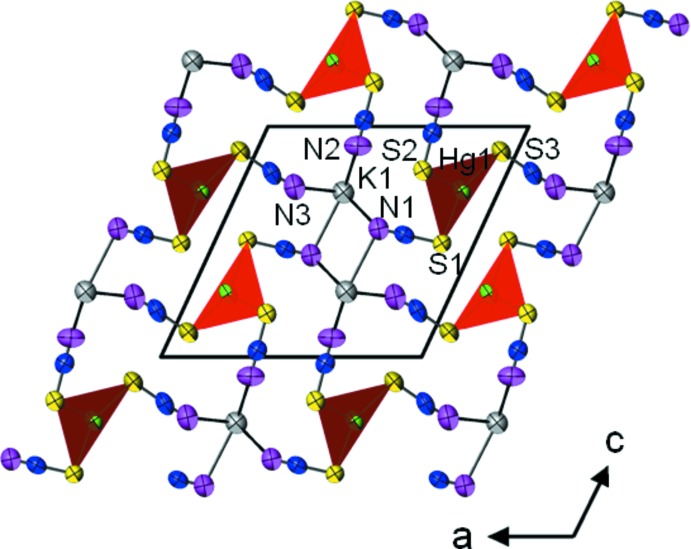
The crystal structure of K[Hg(SCN)3] in a projection along [010]. Displacement ellipsoids are drawn at the 90% probability level. Colour code: Hg green, S yellow, C blue, N magenta, K grey. The HgS4 polyhedron is shown in red.

3 4 . DOI: 10.1107/S1600536814013403/hb0013fig1.tif
The crystal structure of K[Hg(SCN)_3_] in a projection along [010]. Displacement ellipsoids are drawn at the 90% probability level. Colour code: Hg green, S yellow, C blue, N magenta, K grey. The HgS_4_ polyhedron is shown in red.

CCDC reference: 1006909

Additional supporting information:  crystallographic information; 3D view; checkCIF report

## supplementary crystallographic information

### S1. Experimental

Hg(SCN)_2_ (0.5 g) was dissolved under heating in a water–ethanol mixture (1:1 *v*/*v*) to which KSCN (0.1 g) was added. After one week, colourless crystals with a lath-like form were obtained from the remaining solution.

### S2. Refinement

For better comparison with the previous determination by Zhdanov & Sanadze (1952), the original nonreduced cell setting as well as the atom labelling and the atomic coordinates were used as starting parameters for the refinement.

Bowmaker *et al.* (1998) reported a doubled unit cell for K[Hg(SCN)_3_] with *a* = 11.9119 (3), *b* = 4.0201 (1), *c* = 18.7095 (3) Å, β = 91.852 (1)°, *P*2_1_/*n*, *Z* = 4. However, no superstructure reflections were found in the current redetermination at 293 K, while Bowmaker *et al.* (1998) used intensity data measured at 150 K. Therefore it appears likely that K[Hg(SCN)_3_] has a phase transition between these two temperatures. The two unit cells of the room-temperature phase in *P*2_1_/*m* and the low-temperature phase in *P*2_1_/*n* are related by the matix (101,010,101), revealing a *klassengleiche* symmetry reduction of index 2 (Müller, 2013).

The highest and lowest electron densities are found 0.66 and 0.28 Å, respectively, from atom S3.

### Crystal data

**Table:**

K[Hg(NCS)_3_]	*F*(000) = 372
*M**_r_* = 413.93	*D*_x_ = 3.005 Mg m^−^^3^
Monoclinic, *P*2_1_/*m*	Mo *K*α radiation, λ = 0.71073 Å
Hall symbol: -P 2yb	Cell parameters from 4570 reflections
*a* = 11.2727 (11) Å	θ = 3.4–33.7°
*b* = 4.0775 (4) Å	µ = 17.90 mm^−^^1^
*c* = 10.9764 (10) Å	*T* = 293 K
β = 114.951 (4)°	Lath, colourless
*V* = 457.44 (8) Å^3^	0.30 × 0.06 × 0.04 mm
*Z* = 2	

### Data collection

**Table:**

Bruker APEXII CCD diffractometer	3765 independent reflections
Radiation source: fine-focus sealed tube	2358 reflections with *I* > 2σ(*I*)
Graphite monochromator	*R*_int_ = 0.032
ω and φ scans	θ_max_ = 44.4°, θ_min_ = 3.4°
Absorption correction: multi-scan (*SADABS*; Bruker, 2008)	*h* = −19→22
*T*_min_ = 0.226, *T*_max_ = 0.504	*k* = −7→7
11940 measured reflections	*l* = −21→18

### Refinement

**Table:**

Refinement on *F*^2^	Primary atom site location: isomorphous structure methods
Least-squares matrix: full	*w* = 1/[σ^2^(*F*_o_^2^) + (0.0218*P*)^2^ + 0.0583*P*] where *P* = (*F*_o_^2^ + 2*F*_c_^2^)/3
*R*[*F*^2^ > 2σ(*F*^2^)] = 0.028	(Δ/σ)_max_ = 0.001
*wR*(*F*^2^) = 0.062	Δρ_max_ = 1.27 e Å^−^^3^
*S* = 1.03	Δρ_min_ = −1.33 e Å^−^^3^
3765 reflections	Extinction correction: *SHELXL97* (Sheldrick, 2008), Fc^*^=kFc[1+0.001xFc^2^λ^3^/sin(2θ)]^-1/4^
68 parameters	Extinction coefficient: 0.0219 (8)
0 restraints	

### Special details

**Table:**

Geometry. All e.s.d.'s (except the e.s.d. in the dihedral angle between two l.s. planes) are estimated using the full covariance matrix. The cell e.s.d.'s are taken into account individually in the estimation of e.s.d.'s in distances, angles and torsion angles; correlations between e.s.d.'s in cell parameters are only used when they are defined by crystal symmetry. An approximate (isotropic) treatment of cell e.s.d.'s is used for estimating e.s.d.'s involving l.s. planes.
Refinement. Refinement of *F*^2^ against ALL reflections. The weighted *R*-factor *wR* and goodness of fit *S* are based on *F*^2^, conventional *R*-factors *R* are based on *F*, with *F* set to zero for negative *F*^2^. The threshold expression of *F*^2^ > σ(*F*^2^) is used only for calculating *R*-factors(gt) *etc*. and is not relevant to the choice of reflections for refinement. *R*-factors based on *F*^2^ are statistically about twice as large as those based on *F*, and *R*- factors based on ALL data will be even larger.

### Fractional atomic coordinates and isotropic or equivalent isotropic displacement parameters (Å^2^)

**Table:**

	*x*	*y*	*z*	*U*_iso_*/*U*_eq_	
Hg1	0.145383 (12)	0.2500	0.725128 (12)	0.03813 (6)	
K1	0.59433 (8)	0.7500	0.71536 (7)	0.03802 (15)	
S1	0.12455 (7)	0.2500	0.49391 (8)	0.03495 (17)	
S2	0.32808 (7)	0.7500	0.80993 (8)	0.03121 (14)	
S3	0.06157 (10)	0.2500	0.89262 (10)	0.0704 (4)	
C1	0.2848 (3)	0.2500	0.5314 (3)	0.0313 (6)	
C2	0.3579 (3)	0.7500	0.9719 (3)	0.0312 (6)	
C3	0.9019 (4)	0.2500	0.7977 (4)	0.0357 (6)	
N1	0.3940 (3)	0.2500	0.5574 (3)	0.0477 (8)	
N2	0.6210 (4)	0.2500	0.9165 (3)	0.0500 (8)	
N3	0.7909 (4)	0.2500	0.7382 (4)	0.0601 (10)	

### Atomic displacement parameters (Å^2^)

**Table:**

	*U*^11^	*U*^22^	*U*^33^	*U*^12^	*U*^13^	*U*^23^
Hg1	0.03374 (7)	0.05407 (10)	0.03323 (8)	0.000	0.02060 (5)	0.000
K1	0.0377 (3)	0.0421 (4)	0.0364 (3)	0.000	0.0177 (3)	0.000
S1	0.0247 (3)	0.0544 (5)	0.0247 (3)	0.000	0.0094 (3)	0.000
S2	0.0292 (3)	0.0372 (4)	0.0296 (3)	0.000	0.0147 (3)	0.000
S3	0.0341 (4)	0.1516 (14)	0.0307 (4)	0.000	0.0188 (4)	0.000
C1	0.0334 (14)	0.0392 (16)	0.0243 (12)	0.000	0.0150 (11)	0.000
C2	0.0268 (12)	0.0344 (15)	0.0309 (14)	0.000	0.0107 (11)	0.000
C3	0.0376 (15)	0.0406 (17)	0.0398 (16)	0.000	0.0269 (13)	0.000
N1	0.0329 (14)	0.075 (2)	0.0379 (15)	0.000	0.0175 (12)	0.000
N2	0.0529 (19)	0.064 (2)	0.0292 (14)	0.000	0.0134 (13)	0.000
N3	0.0394 (17)	0.096 (3)	0.050 (2)	0.000	0.0248 (15)	0.000

### Geometric parameters (Å, º)

**Table:**

Hg1—S3	2.3954 (11)	K1—K1^iii^	4.7485 (14)
Hg1—S1	2.4481 (8)	S1—C1	1.675 (3)
Hg1—S2	2.7653 (6)	S2—C2	1.664 (3)
Hg1—S2^i^	2.7653 (6)	S2—Hg1^ii^	2.7653 (6)
K1—N2^ii^	2.926 (2)	S3—C3^iv^	1.657 (4)
K1—N2	2.926 (2)	C1—N1	1.140 (4)
K1—N3^ii^	2.943 (3)	C1—K1^iii^	3.508 (3)
K1—N3	2.943 (3)	C2—N2^v^	1.145 (5)
K1—N1	2.993 (2)	C3—N3	1.141 (6)
K1—N1^ii^	2.993 (2)	C3—S3^vi^	1.657 (4)
K1—N1^iii^	3.051 (3)	N1—K1^i^	2.993 (2)
K1—C1^iii^	3.508 (3)	N1—K1^iii^	3.051 (3)
K1—S2	3.5657 (12)	N2—C2^v^	1.145 (5)
K1—K1^i^	4.0775 (4)	N2—K1^i^	2.926 (2)
K1—K1^ii^	4.0775 (4)	N3—K1^i^	2.943 (3)
			
S3—Hg1—S1	154.06 (3)	C1^iii^—K1—K1^i^	90.0
S3—Hg1—S2	102.74 (2)	S2—K1—K1^i^	90.0
S1—Hg1—S2	94.65 (2)	N2^ii^—K1—K1^ii^	45.83 (5)
S3—Hg1—S2^i^	102.74 (2)	N2—K1—K1^ii^	134.17 (5)
S1—Hg1—S2^i^	94.65 (2)	N3^ii^—K1—K1^ii^	46.15 (5)
S2—Hg1—S2^i^	95.00 (2)	N3—K1—K1^ii^	133.85 (5)
N2^ii^—K1—N2	88.34 (9)	N1—K1—K1^ii^	132.94 (4)
N2^ii^—K1—N3^ii^	67.60 (10)	N1^ii^—K1—K1^ii^	47.06 (4)
N2—K1—N3^ii^	125.76 (10)	N1^iii^—K1—K1^ii^	90.0
N2^ii^—K1—N3	125.76 (10)	C1^iii^—K1—K1^ii^	90.0
N2—K1—N3	67.60 (10)	S2—K1—K1^ii^	90.0
N3^ii^—K1—N3	87.70 (10)	K1^i^—K1—K1^ii^	180.00 (5)
N2^ii^—K1—N1	136.39 (10)	N2^ii^—K1—K1^iii^	155.60 (6)
N2—K1—N1	76.98 (8)	N2—K1—K1^iii^	108.20 (5)
N3^ii^—K1—N1	151.42 (11)	N3^ii^—K1—K1^iii^	112.78 (8)
N3—K1—N1	86.24 (8)	N3—K1—K1^iii^	78.02 (8)
N2^ii^—K1—N1^ii^	76.98 (8)	N1—K1—K1^iii^	38.66 (6)
N2—K1—N1^ii^	136.39 (10)	N1^ii^—K1—K1^iii^	78.70 (6)
N3^ii^—K1—N1^ii^	86.24 (7)	N1^iii^—K1—K1^iii^	37.79 (4)
N3—K1—N1^ii^	151.42 (11)	C1^iii^—K1—K1^iii^	52.26 (4)
N1—K1—N1^ii^	85.87 (8)	S2—K1—K1^iii^	102.20 (3)
N2^ii^—K1—N1^iii^	135.00 (5)	K1^i^—K1—K1^iii^	64.574 (8)
N2—K1—N1^iii^	135.00 (5)	K1^ii^—K1—K1^iii^	115.426 (8)
N3^ii^—K1—N1^iii^	75.01 (9)	C1—S1—Hg1	97.08 (11)
N3—K1—N1^iii^	75.01 (9)	C2—S2—Hg1	98.38 (7)
N1—K1—N1^iii^	76.45 (8)	C2—S2—Hg1^ii^	98.38 (7)
N1^ii^—K1—N1^iii^	76.45 (8)	Hg1—S2—Hg1^ii^	95.00 (2)
N2^ii^—K1—C1^iii^	129.27 (7)	C2—S2—K1	119.70 (11)
N2—K1—C1^iii^	129.27 (7)	Hg1—S2—K1	120.06 (2)
N3^ii^—K1—C1^iii^	62.76 (8)	Hg1^ii^—S2—K1	120.06 (2)
N3—K1—C1^iii^	62.76 (8)	C3^iv^—S3—Hg1	101.12 (13)
N1—K1—C1^iii^	89.79 (8)	N1—C1—S1	179.8 (3)
N1^ii^—K1—C1^iii^	89.79 (8)	N1—C1—K1^iii^	57.5 (2)
N1^iii^—K1—C1^iii^	18.38 (8)	S1—C1—K1^iii^	122.68 (14)
N2^ii^—K1—S2	67.14 (8)	N2^v^—C2—S2	179.7 (3)
N2—K1—S2	67.14 (8)	N3—C3—S3^vi^	176.4 (4)
N3^ii^—K1—S2	132.15 (6)	C1—N1—K1	128.00 (14)
N3—K1—S2	132.15 (6)	C1—N1—K1^i^	128.00 (14)
N1—K1—S2	69.32 (6)	K1—N1—K1^i^	85.87 (8)
N1^ii^—K1—S2	69.32 (6)	C1—N1—K1^iii^	104.1 (2)
N1^iii^—K1—S2	132.49 (6)	K1—N1—K1^iii^	103.55 (8)
C1^iii^—K1—S2	150.86 (6)	K1^i^—N1—K1^iii^	103.55 (8)
N2^ii^—K1—K1^i^	134.17 (5)	C2^v^—N2—K1^i^	135.73 (5)
N2—K1—K1^i^	45.83 (5)	C2^v^—N2—K1	135.73 (5)
N3^ii^—K1—K1^i^	133.85 (5)	K1^i^—N2—K1	88.34 (9)
N3—K1—K1^i^	46.15 (5)	C3—N3—K1^i^	130.79 (15)
N1—K1—K1^i^	47.06 (4)	C3—N3—K1	130.79 (15)
N1^ii^—K1—K1^i^	132.94 (4)	K1^i^—N3—K1	87.70 (10)
N1^iii^—K1—K1^i^	90.0		

Symmetry codes: (i) *x*, *y*−1, *z*; (ii) *x*, *y*+1, *z*; (iii) −*x*+1, −*y*+1, −*z*+1; (iv) *x*−1, *y*, *z*; (v) −*x*+1, −*y*+1, −*z*+2; (vi) *x*+1, *y*, *z*.

## References

[bb1] Bowmaker, G. A., Churakov, A. V., Harris, R. K., Howard, J. A. K. & Apperley, D. C. (1998). *Inorg. Chem.* **37**, 1734–1743.

[bb2] Bruker (2008). *APEX2*, *SAINT* and *SADABS* Bruker AXS Inc., Madison, Wisconsin, USA.

[bb3] Dowty, E. (2006). *ATOMS for Windows* Shape Software, Kingsport, Tennessee, USA.

[bb4] Müller, U. (2013). In *Symmetry Relationships between Crystal Structures* Oxford University Press.

[bb5] Sheldrick, G. M. (2008). *Acta Cryst.* A**64**, 112–122. 10.1107/S0108767307043930 18156677

[bb6] Westrip, S. P. (2010). *J. Appl. Cryst.* **43**, 920–925.

[bb7] Zhdanov, G. S. & Sanadze, V. V. (1952). *Zh. Fiz. Khim.* **26**, 469–478.

