# Supplementary figures and images for: Crystal structure of K[Hg(SCN)3] – a redetermination
Source: Acta Crystallogr Sect E Struct Rep Online. 2014 Aug 1;70(Pt 9):i46. doi: 10.1107/S1600536814013403 (PMC4186170; doi:10.1107/S1600536814013403)

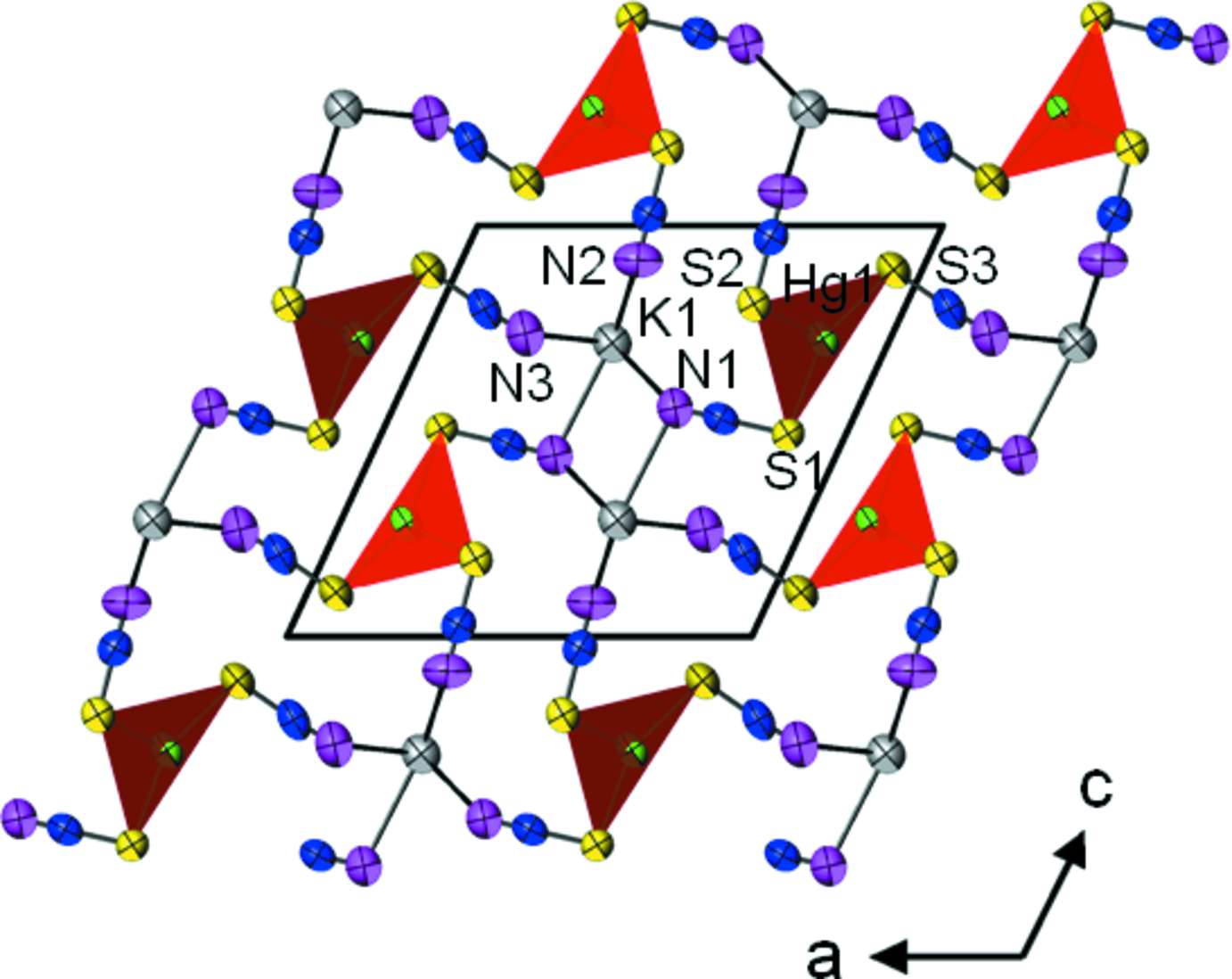

Supplement: Supplementary file 3 [file e-70-00i46-fig1.tif]
